# Brown Adipose Tissue in Humans Is Activated by Elevated Plasma Catecholamines Levels and Is Inversely Related to Central Obesity

**DOI:** 10.1371/journal.pone.0021006

**Published:** 2011-06-20

**Authors:** Qidi Wang, Min Zhang, Guang Ning, Weiqiong Gu, Tingwei Su, Min Xu, Biao Li, Weiqing Wang

**Affiliations:** 1 Shanghai Key Laboratory for Endocrine Tumors and Key Laboratory for Endocrine and Metabolic Diseases of Chinese Health Ministry, Rui-Jin Hospital, Shanghai Jiao-Tong University School of Medicine, Shanghai, China; 2 Shanghai Clinical Center for Endocrine and Metabolic Diseases and Division of Endocrinology and Metabolism of The E-Institute of Shanghai Universities, Rui-Jin Hospital, Shanghai Jiao-Tong University School of Medicine, Shanghai, China; 3 Department of Nuclear Medicine, Rui-Jin Hospital, Shanghai Jiao-Tong University School of Medicine, Shanghai, China; Cardiff University, United Kingdom

## Abstract

**Background:**

Recent studies have shown that adult human possess active brown adipose tissue (BAT), which might be important in controlling obesity. It is known that ß-adrenoceptor-UCP1 system regulates BAT in rodent, but its influence in adult humans remains to be shown. The present study is to determine whether BAT activity can be independently stimulated by elevated catecholamines levels in adult human, and whether it is associated with their adiposity.

**Methodology/Principal Findings:**

We studied 14 patients with pheochromocytoma and 14 normal subjects who had performed both ^18^F-fluorodeoxyglucose positron emission tomography/computed tomography (^18^F-FDG PET/CT) and plasma total metanephrine (TMN) measurements during 2007–2010. The BAT detection rate and the mean BAT activity were significantly higher in patients with elevated TMN levels (Group A: 6/8 and 6.7±2.1 SUVmean· g/ml) than patients with normal TMN concentrations (Group B: 0/6 and 0.4±0.04 SUVmean· g/ml) and normal subjects (Group C: 0/14 and 0.4±0.03 SUVmean·g/ml). BAT activities were positively correlated with TMN levels (R = 0.83, p<0.0001) and were inversely related to body mass index (R = −0.47, p = 0.010), visceral fat areas (R = −0.39, p = 0.044), visceral/total fat areas (R = −0.52, p = 0.0043) and waist circumferences (R = −0.43, p = 0.019). Robust regression revealed that TMN (R = 0.81, p<0.0001) and waist circumferences (R = −0.009, p = 0.009) were the two independent predictors of BAT activities.

**Conclusions/Significance:**

Brown adipose tissue activity in adult human can be activated by elevated plasma TMN levels, such as in the case of patients with pheochromocytoma, and is negatively associated with central adiposity.

## Introduction

Mammals have two types of adipose tissue that control whole-body energy metabolism, the well-understood white adipose tissue for energy storage [1] and brown adipose tissue (BAT) for cold- and diet-induced thermogenesis [2]. BAT thermogenesis is dependent on the ß-adrenergically mediated activation of lipolysis and subsequent degradation of fatty acids via uncoupling protein 1 (UCP1) that dissipate large amounts of chemical energy as heat [3]. Brown adipose tissue is most obvious in small mammals and infant humans, but was often believed to be lost postnatally within the first few years of human life [2]. Recent studies using positron emission tomography and computed tomography (PET/CT) have demonstrated that healthy adult humans do possess significant depots of metabolically active BAT [4], [5], [6], which is acutely cold-induced [5], [6] and is inversely correlated with body mass index (BMI) and age [4], [5]. Although several BAT-influencing factors have been identified [4], [5], [6], the role of ß-adrenoceptor-UCP1 system- which is functional in rodents in regulating BAT activity [2]- has not yet been fully studied in adult humans. In rodents, it is found that the sensation of cold causes sympathetic nerve terminals in the brown adipose tissue to release catecholamines that stimulate BAT proliferation and activation [2]. In addition, brown fat activity can be activated by ß-adrenergic activator or catecholamines, and causes the emergence of UCP-1 expressing BAT in classic depots of white fat [7], [8]. In light of these observations, we compared BAT activities, long-lived catecholamine metabolites, i.e. plasma metanephrine and normetanephrine concentrations, as well as body fat parameters in normal subjects and patients with pheochromocytoma, a neuroendocrine tumor intermittently secretes excessive amounts of catecholamines. Our present data demonstrate that BAT activity is strongly and positively correlated with catecholamines concentrations in the subjects, and is negatively related with their adiposity, especially waist circumferences; suggesting an important role of adrenoceptor-UCP1 system in the regulation of active BAT and subsequent central obesity in adult human.

## Methods

### Ethics statement

This study was approved by the Institutional Review Board of the Rui-jin Hospital Affiliated to Shanghai Jiao-Tong University School of Medicine and was in accordance with the principle of the Helsinki Declaration II. The written informed consent was obtained from each participant.

### Subjects

From all pheochromocytoma patients diagnosed during 2007–2010 at Shanghai Clinical Center for Endocrine and Metabolic Diseases, fourteen have performed consecutive ^18^F-fluorodeoxyglucose (^18^F-FDG) PET/CT whole-body scans. These patients were confirmed by postoperative pathology. Fourteen age- and BMI-matched healthy volunteers were also recruited for the study. Data on age, sex, weight, height, fasting glucose, triglyceride, cholesterol and thyroid hormone levels, medication use were obtained for all subjects.

In the present study, plasma catecholamines level was represented by its long-lived metabolic products, i.e. plasma metanephrine (MN) and normetanephrine (NMN) created by action of catechol-O-methyl transferase on epinephrine and norepinephrine, determined by reverse-phase high-performance liquid chromatography (HPLC) (Agilent 1100 series, Santa Clara USA) with electrochemical detection (ESA-A Dionex Company, Chelmsford USA) as previously described [9], [10]. In our earlier study on 40 healthy volunteers, plasma concentrations of MN ranged between 18.8 and 78.2 ng/L (mean 39.9±16.6 ng/L), and NMN between 17.5 and 118.8 ng/L (mean 61.1±25.6 ng/L). Thus, using the upper 2SD as cut-off points, the subjects can be classified into three groups: 8 pheochromocytoma patients with high total metanephrine (TMN: MN+NMN) (>185 ng/L, Group A), 6 pheochromocytoma patients with normal TMN (<185 ng/L, Group B) and 14 normal controls (Group C).

### Brown adipose tissue activity by PET/CT scan

All of the subjects were studied in the morning after an overnight fast beginning around 10 p.m. the night before. The blood glucose concentration of each subject was controlled under the level of 7.4 mmol/l before ^18^F-FDG (4.44–5.55 MBq/kg) was injected intravenously. They were kept under thermoneutral conditions (23–25°C) for at least 1 h after injection. Later the ^18^F-FDG PET/CT scans were performed with a GE Discovery STE16 integrated PET/CT scanner combining the ability to acquire CT images and PET data of the same subject in one session. The whole-body CT data were acquired by a continuous spiral technique on a 16-slice helical CT (gantry rotation speed, 0.8 s per rotation, 140 KV, 17.5 mm per rotation table speed). All CT scans were obtained with 3.75 mm thick axial sections and the axial field of view was 15.6 cm. Subsequently, a positron emission scan was performed from the thigh to the head at a 3 min/bed position speed. The attenuation-corrected PET images, combined with CT data, were reconstructed by an ordered-subset expectation maximization algorithm.

PET and CT images were coregistered and analyzed by a GEAW workstation with PET/CT Volume Viewer software. Two experienced blinded observers assessed the FDG uptake, particularly in both sides of the neck and paravertebral regions, by visually judging the radioactivity greater than background [1]. In parallel, the activity of BAT was quantified by mean standardized uptake values (SUVmean · g/ml) [4] which is an automatic method based on GE AW workstation [11]. The standardized uptake values, defined as the activity per milliliter within the region of interest divided by the injected dose in megabecquerels per kilogram of body weight was determined [4]. Calculations were performed using PET/CT Volume Viewer software.

### Visceral/subcutaneous fat areas and waist circumferences

Abdominal fat distribution was examined at the umbilicus level in the supine position using CT, according to the previously described procedure [12]. The intra-abdominal visceral fat areas (VFA), subcutaneous fat areas (SFA), and waist circumferences (WC) were measured with image analysis software package (Fat scan, N2 system, Osaka, Japan). Visceral/total fat areas (V/T) was calculated as VFA/(VFA+SFA) ×100%.

### Statistics

Statistical analysis was performed with SAS 9.2 (SAS Institute, Cary NC). All continuous parameters were summarized as means±SD, and all categorical parameters were summarized as proportions. To compare between-group differences, robust ANOVA was performed to compare difference in BAT activity between groups [13]. A conventional linear regression model was used to explore the association between BAT activity and other parameters, such as TMN, BMI, VFA, SFA, V/T and WC. To minimize the impact of outliers on regression estimators, robust regression analysis was performed to identify factors that are independently associated with BAT [13]. The P-values reported were two-sided. A P-value of less than 0.05 indicated statistical significance.

## Results

Information on all pheochromocytoma patients to Shanghai Clinical Center for Endocrine and Metabolic Diseases between 2007 and 2010 was recorded and collated on an intra-hospital database. This database contained information on clinical details, biochemical examination, imaging records and pathology results. Based on this database, we extracted records of all patients with pheochromocytoma that have performed PET/CT whole-body scans. All of the patients presented clinical findings of hypertension, associated with positive results in PET/CT, and finally confirmed by post-operative pathology. Among them, eleven cases (80%) were adrenal pheochromocytoma and three cases (20%) were from extra-adrenal chromaffin tissues. Two patients were reported as metastatic disease. Plasma total metanephrine (TMN) levels were significantly higher in eight patients with pheochromocytoma (Group A: 2403±1189 ng/L, Table 1) than the other 6 patients (Group B: 122±12 ng/L, Table 1) and normal subjects (Group C: 116.4±8.1 ng/L, Table 1). Serum fasting glucose, triglyceride, cholesterol and thyroid hormone levels (FT3, FT4) were comparable within three groups (Table 1). None of the subjects have taken ß -adrenergic blockers at the time of PET scan.

**Table 1:** Characteristics of the subjects.

Characteristic	Pheochromocytoma patients		Normal subjects
	High TMN(Group A, N = 8)	Normal TMN(Group B, N = 6)	Normal TMN(Group C, N = 14)
Age (Yr)	37.8±4.9	45.8±4.8	46.9±2.4
Detectable Brown-adipose-tissue	6/8	0/6	0/14
Brown-adipose-tissue# ###activity (SUV_mean_·g/ml)#	6.7±2.1	0.4±0.04*	0.4±0.03*
Total metanephrines ˆ(TMN, ng/L) ˆ	2403±1189	122±12*	116±8.1*
Body mass index†	22.4±1.0	23.6±1.6	23.9±0.6
Visceral Fat Areas‡	50.7±9.2	91.5±34.3	85.7±13.6
Subcutaneous Fat areas‡	161.5±15.5	217.5±37.1	127.1±12.3
Waist circumferences‡	83.8±3.3	93.6±5.7	86.4±2.0
Fasting blood glucose (mM)	5.6±0.5	5.3±0.4	5.3±0.2
Triglyceride (mM)	1.3±0.3	1.3±0.2	1.5±0.3
Cholesterol (mM)	4.7±0.2	4.8±0.5	4.4±0.3
Free T3 (pM)	4.1±0.2	3.9±0.4	4.0±0.2
Free T4 (pM)	12.5±0.5	12.3±0.7	13.5±0.5

# Brown adipose tissues activity was quantified by mean standardized uptake values (SUVmean · g/ml) by PET/CT.

ˆ Total metanephrines were calculated as metanephrine (MN) plus normetanephrine (NMN).

† Body mass index was calculated as weight in kilograms divided by height in meters squared.

‡ Visceral fat areas, subcutaneous fat areas and waist circumferences were examined at the umbilicus level in the supine position using CT.

*compared to pheochromocytoma patients with high TMN (Group A), p<0.001.

**Figure 1:**
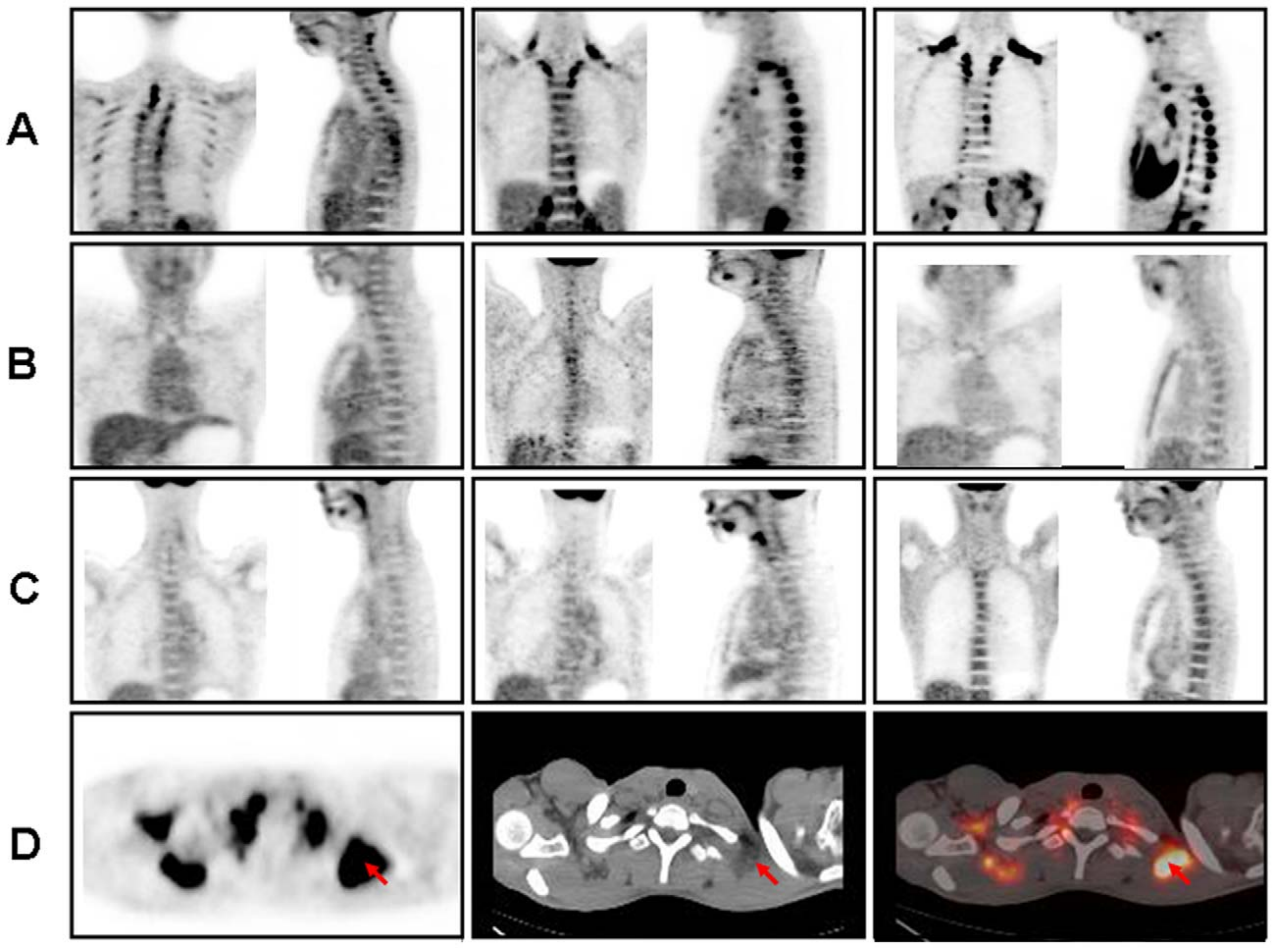
BAT activity can be detected by PET/CT in pheochromocytoma patients with elevated TMN concentration. The results of PET–CT scanning in pheochromocytoma patients were compared to healthy subjects. Panels A-C shows images of the neck and upper thoracic region of individuals with variable physiologic uptake and distribution of ^18^F-fluorodeoxyglucose (^18^F-FDG) in adipose tissue. A front and a side scan were shown individually. The images in row (A) are from three pheochromocytoma patients with high total metanephrine (TMN) levels (Group A). Three pheochromocytoma patients with normal TMN concentrations from Group B and three healthy subjects from Group C were shown in row (B) and (C) accordingly. A PET scan in the transverse plane (Panel D, left) shows the areas of brown adipose tissue (arrow), and a CT scan (Panel D, middle) confirms the areas of brown adipose tissue (arrow) according to fat density and location. Fusion of the PET and CT scans (Panel D, right) shows that ^18^F-FDG uptake is localized in fatty tissue (arrow).

On the fused PET/CT images, the brown adipose tissue was located not in muscle tissue but in fat tissue, within the neck, supraclavicular and paravertebral region (Fig. 1D) as previously reported [4], [5], [6]. Six out of eight patients from Group A had evidence of brown adipose tissue (Figure 1A, Table 1). Only two patients had no ^18^F-FDG uptake in areas of adipose tissue. These two subjects either had the highest BMI, total fat areas or lowest TMN levels. On the contrary, there was no detectable brown adipose tissue in any of the subjects with normal TMN concentrations irrespective of their pheochromocytoma disease background (Fig. 1B-C, Table 1). Similar results were observed when the mean BAT activity was taken into account: it was significantly higher in Group A patients (6.7±2.1 SUVmean· g/ml)than that in Group B (0.4±0.04 SUVmean· g/ml, p<0.0001) and Group C (0.4±0.03 SUVmean·g/ml, p<0.0001) (Table 1). No significant difference in BAT activity was observed between Group B and Group C that presented comparable TMN levels (Table 1). These data indicate that circulating catecholamines level, rather than the disease itself, might determine BAT activity in pheochromocytoma patients.

**Figure 2:**
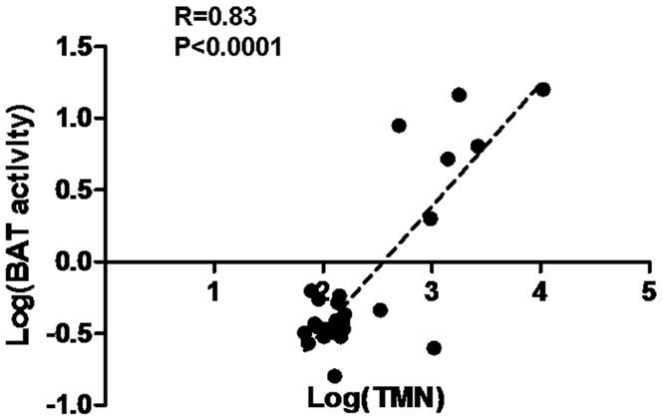
Activities of brown adipose tissue in relation to total metanephrine levels. For all subjects, the activities of brown-adipose-tissue (BAT) were plotted against their total metanephrine (TMN) values following Log transformation. Statistical analysis was performed using Pearson correlation test: R = 0.83, p<0.0001.

We further performed a conventional linear regression to explore the association between BAT activities and TMN concentrations in our subjects. For the first time, we demonstrated a strong and significantly positive correlation between BAT activities and TMN levels (both in log scale; R = 0.83, p<0.0001, Fig. 2). These results indicate that BAT activity can be strongly induced by increasing adrenergic input in adult humans, such as in the case of patients with pheochromocytoma.

It has been reported that the amount of brown adipose tissue is inversely related to body mass index (BMI) in adult human [4], [6]. In the present study, we included not only BMI but also other parameters, such as visceral fat areas, subcutaneous fat areas, visceral/total fat areas and waist circumferences, which measured abdominal fat volume and distribution [12]. Pearson correlation test revealed that LogBAT was negatively correlated with BMI (R = −0.47, p = 0.010), visceral fat areas (R = −0.39, p = 0.044), visceral/total fat areas (R = −0.52, p = 0.0043), waist circumferences (R = −0.43, p = 0.019), but not with subcutaneous fat areas (R = −0.15, p = 0.45). In the next step, robust regression analysis was performed to identify factors that were independently associated with BAT (log scale) [13]. Among age, sex, BMI, VFA, SFA, V/T, WC, and TMN examined, only TMN (R = 0.81, p<0.0001) and waist circumferences (R = −0.009, p = 0.009) were found independently associated with BAT activity (Table 2).

**Table 2:** Robust regression analysis to identify factors that are independently associated with brown-adipose-tissue activity.

Factor	Initial model		Final model	
	β±SE	P-value	β±SE	P-value
Age	0.002±0.004	0.61		
Male sex	0.04±0.09	0.72		
BMI	−0.010±0.026	0.67		
VFA	0.0002±0.002	0.93		
SFA	0.001±0.002	0.63		
WC	−0.02±0.01	0.20	−0.009±0.003	0.009
V/T	0.74±0.96	0.44		
Log (TMN)	0.91±0.09	<0.0001	0.81±0.06	<0.0001

Robust regression analysis was performed to identify factors that are independently associated with brown-adipose-tissue (BAT, log scale). An initial robust regression model included age, sex, body mass index (BMI), visceral fat areas (VFA), subcutaneous fat areas (SFA), waist circumferences (WC), visceral/total fat areas (V/T) and log (TMN). Those variables that did not make significant contributions to the models were deleted in a stage-wise manner, yielding the final models. The final model is shown including 2 significant predictors, Log(TMN) and waist circumferences.

## Discussion

It was normally believed that brown fat is present only in fetuses and infants and diminishes in adults [2], however recent studies have clearly demonstrated that adults do possess metabolically active brown fat which can be detected by PET/CT imaging and be stimulated by cold exposure [4], [5], [6]. Moreover, the observation that the amount of BAT is inversely correlated with body mass index [1], [4], [5], have led to an increased interest in the potential importance of BAT in regulating body weight and as a potential target for treating obesity [1], [4], [5], [6]. With this knowledge of BAT, targeted therapies are under study, by either induction of already available BAT (with ß-adrenergic activator) [14], [15] or changing the genetic structure for differentiating tissue from the preadipocyte phase to BAT [16], [17].

From studies performed in vitro, catecholamines have been suggested to play a prime role modulating not only brown preadipocyte proliferation, mature brown adipocytes differentiation, but also their apoptosis [2]. The present study now shows that the circulating total metanephrines, long-lived catecholamines metabolites levels and BAT activities were simultaneously elevated in patients with pheochromocytoma under thermoneutral conditions. These data suggest that the catecholamines such as epinephrine and norepinephrine might stimulate BAT activity, which is supported by the following evidence. First, 6 of 14 patients with pheochromocytoma showed a substantial FDG uptake into adipose tissue of the supraclavicular and paraspinal regions, whereas no detection was found in normal controls. Second, all the BAT positive patients were presenting elevated TMN levels, while none of the patients with normal TMN had brown adipose tissue detections, indicating that BAT activity is hormone-dependent, rather than disease-specific. Third, we were the first to provide a strong and positive correlation between BAT activity and circulating TMN concentration (r = 0.83, p<0.0001), thus undoubtedly demonstrated that BAT activity could be activated adrenergically in humans. Fourth, TMN was the strongest predictor of BAT activity, independent of other influencing factors such as age, sex, BMI, and fat distribution. The present finding is consistent with several earlier case reports by nuclear medicine physicians: an increased glucose uptake was observed in a 50-year-old male patient with adrenal pheochromcytoma [18] and a disappearance of prominent FDG uptake in BAT was found in a 25-year-old woman with mediastinal pheochromocytoma after the tumor resection [19]. A more detailed finding by Hadi et al reported 26 (27.0%) of 96 patients with known or suspected pheochromocytoma showed BAT evaluated with (18)F-FDA or (18)F-FDG PET/CT [20]. However, these studies failed to further investigate the direct relationship between catecholamines levels and BAT activation. Taken together, brown fat in adult human can be activated and can expand quite massively due to catecholamines over-secretion, proving a central role of adrenergic activation in BAT regulation.

It is well established that catecholamines-mediated effects on energy expenditure and alterations in the efficiency of carbohydrate metabolism are possible mediators of changes in body weight. Catecholamines can increase fat lipolysis and reduce adipogenesis or the creation of new fat [21] and this effect is attenuated in the subcutaneous adipose tissue of obese subjects [22], [23]. Our finding that catecholamines-associated brown adipose tissue activation might provide additional explanation on the impact of these hormones on body weight control. Early evidence for BAT as a tissue affecting adiposity in rodents came from animals with surgically denervated interscapular BAT [24] or transgenic mice with 60–70% reduction in BAT mass [25], [26]: these animals accumulated abnormal amounts of body fat. Consistent with several recent studies on adult human [1], [4], [5], our data found an inverse relation between BAT activity and adiposity. Considering a key role of adrenergic input on BAT regulation, it is relevant in our study that brown adipose tissue, which is activated by catecholamines, results in burning large amounts of abdominal fat through oxidation in mitochondria. In addition, catecholamines-induced trans-differentiation of existing white fat cells to brown adipocytes [2] can not be neglected. In mice and rats, exposure to cold or ß-adrenergic agonist induces the appearance of brown adipocytes in traditional white fat pads [27], [28], [29]. In patients with pheochromocytoma, the induction of UCP-1 expression was observed in intra-abdominal adipose tissue [30], [31]. Although some plasticity seems to exist between the white and brown adipocytes, the extent of such shift in humans and the molecular mechanisms governing these remain unknown. Another interesting point to be noted is that by using abdominal fat areas measurement, we found that BAT was inversely related with central obesity parameters, i.e. visceral fat areas, visceral/total fat areas, waist circumferences of the subjects. On the contrary, subcutaneous fat areas did not show significant correlation. Moreover, by robust stepwise regression we identified waist circumferences, a parameter of central obesity [32], one of the two significant and independent predictors of BAT activity besides TMN. It has been reported that waist circumference correlates highly with visceral mass and has been shown to predict metabolic risks and coronary heart disease better than other indices of adiposity including BMI [33]. Based on the present observations, we propose that brown adipose tissue might play a more pronounced role on protecting central obesity and reducing metabolic syndrome. Studies can thus be planned to investigate the impact of BAT activity on central obesity and metabolic risk in larger population.

In summary, in the present study, the use of ^18^F-FDG-PET/CT shows that brown adipose tissue is strongly activated in adult human with elevated circulating catecholamines concentration. Waist circumferences preferentially than BMI, is inversely correlated with the amount of brown adipose tissue, suggesting a possible role of brown adipose tissue in protecting against central obesity. Our result may highlight again adrenoceptor-UCP1 system in pharmacological approach to obesity treatment, which has repeatedly been confirmed in experimental animals [14], [15], [34], [35], but not yet approved in humans. The recent evidence that there is a significant amount of brown adipose tissue expressing ß 3-adrenergic receptor in many adult humans, and our current finding that their activity can indeed be stimulated by adrenergic input and is inversely related with visceral fat volume, might offer new hope in searching for intriguing target for the control of whole-body energy balance and obesity.
